# A tomato HD-Zip homeobox protein, LeHB-1, plays an important role in floral organogenesis and ripening

**DOI:** 10.1111/j.1365-313X.2008.03505.x

**Published:** 2008-05-27

**Authors:** Zhefeng Lin, Yiguo Hong, Mingan Yin, Chunyang Li, Ke Zhang, Don Grierson

**Affiliations:** 1 Plant Sciences Division, School of Biosciences, University of NottinghamSutton Bonington Campus, Loughborough, LE12 5RD, UK; 2 Warwick HRI, University of WarwickWellesbourne, Warwick CV35 9EF, UK,; 3 College of Horticulture, Northwest A & F UniversityYangling, Shaanxi 712100, China

**Keywords:** LeHB-1, HD-Zip homeobox protein, LeACO1, floral organogenesis, ripening, tomato

## Abstract

Ethylene is required for climacteric fruit ripening. Inhibition of ethylene biosynthesis genes, 1-aminocyclopropane-1-carboxylate (ACC) synthase and ACC oxidase, prevents or delays ripening, but it is not known how these genes are modulated during normal development. LeHB-1, a previously uncharacterized tomato homeobox protein, was shown by gel retardation assay to interact with the promoter of *LeACO1*, an ACC oxidase gene expressed during ripening. Inhibition of *LeHB-1* mRNA accumulation in tomato fruit, using virus-induced gene silencing, greatly reduced *LeACO1* mRNA levels, and inhibited ripening. Conversely, ectopic overexpression of *LeHB-1* by viral delivery to developing flowers elsewhere on injected plants triggered altered floral organ morphology, including production of multiple flowers within one sepal whorl, fusion of sepals and petals, and conversion of sepals into carpel-like structures that grew into fruits and ripened. Our findings suggest that LeHB-1 is not only involved in the control of ripening but also plays a critical role in floral organogenesis.

## Introduction

The gaseous hormone ethylene regulates many aspects of plant growth and development, including ripening, senescence, abscission, and responses to biotic and abiotic stresses (Abeles *et al.*, 1992). Ethylene biosynthesis occurs via the Yang pathway (Yang and Hoffmann, 1984) using two key biosynthetic enzymes, 1-aminocyclopropane-1-carboxylate (ACC) synthase (ACS) and ACC oxidase (ACO) (Kende, 1993), encoded by differentially expressed multigene families (Barry *et al.*, 1996; Holdsworth *et al.*, 1988; Zarembinski and Theologis, 1994). Antisense-mediated RNA silencing of *LeACS2* prevents tomato ripening (Oeller *et al.*, 1991), and inhibition of *LeACO1* also causes delayed ripening and leaf senescence (Blume and Grierson, 1997; Giovannoni, 2002; Picton *et al.*, 1993). How transcription of the ethylene biosynthesis genes is regulated is therefore critical for our understanding of processes such as ripening, senescence, abscission and responses to stress.

Analysis of the *LeACO1* promoter (−1 to −1855 nucleotides) (accession no: X58732; Blume and Grierson, 1997; ZL and DG, unpublished data) revealed that it contains putative homeobox *cis*-elements, similar to those to which AtHB-1, a class-I homeodomain leucine zipper (HD-Zip) protein from Arabidopsis binds (Sessa *et al.*, 1993). HD-Zip homeobox proteins are defined by the conserved homeodomain (HD) and adjacent leucine zipper motifs (Sessa *et al.*, 1993). They are unique to plants, but are related to other eukaryotic HD proteins (Henriksson *et al.*, 2005). Homeobox genes contain a highly conserved homeobox DNA sequence of 180 bp, encoding a protein which folds into a characteristic DNA binding structure of helix-loop-helix-turn-helix, and are involved in developmental processes. Some HD proteins from both animals and plants have been shown to regulate hormone genes. The homeodomain transcription factors Hesx1 and Prop-1 in mammals, for example, are heavily involved in pituitary organogenesis, and both proteins synergistically regulate the follicle stimulating hormone β subunit gene by binding its promoter region from −852 to −746 bp (Susa *et al.*, 2007). In plants, the knotted-like homeobox (KNOX) proteins function in shoot apical meristems through regulating the production of gibberellin (GA) and cytokinin (Ori *et al.*, 1999). In both Arabidopsis and tobacco, the KNOX proteins directly repress transcription of genes encoding GA 20-oxidases, the enzymes that encode the last step in GA biosynthesis (Hay *et al.*, 2002; Jasinski *et al.*, 2002; Sakamoto *et al.*, 2001).

Since the discovery of KNOTTED1 in maize (Sinha *et al.*, 1993; Vollbrecht *et al.*, 1991), a large number of plant genes encoding HDs have been isolated, and can be classified into six families, depending on their gene structures, sequences, size, HD location and association with other domains (Ariel *et al.*, 2007). These include HD-Zip (homeodomain associated with a leucine zipper), PHD finger (plant homeodomain associated with a finger domain), Bell (named after the distinctive Bell domain), ZF-HD (zinc finger associated with a homeodomain), WOX (wuschel related homeobox) and KNOX (knotted related homeobox) (Ariel *et al.*, 2007; Henriksson *et al.*, 2005).

The HD-Zip family consists of a large number of members. In the Arabidopsis genome, there are 47 HD-Zip proteins that can be divided into four groups, I–IV, according to distinctive features of DNA-binding specificities, gene structures, additional common motifs and physiological functions (Ariel *et al.*, 2007; Henriksson *et al.*, 2005). The HD of HD-Zip transcription factors is responsible for the specific binding to DNA, whereas the Zip domain acts as a dimerization motif. HD-Zip proteins bind to DNA as dimers, and the absence of the Zip domain abolishes the binding activity. Proteins in each group recognize different DNA sequences *in vitro*. Class-I AtHB-1 and class-II AtHB-2, for example, bind to 9-bp DNA sequences with dyad symmetry, CAAT(A/T)ATTG and CAAT(G/C)ATTG, respectively, through the combined HD-Zip domains (Sessa *et al.*, 1993).

Although the HD-Zip proteins have the conserved HD and Zip motifs, their sequences elsewhere are very diverse. The class-I and -II HD-Zip proteins are in general smaller (200–300 aa) than the class-III and -IV proteins (700–800 aa) (Ariel *et al.*, 2007; Henriksson *et al.*, 2005). HD-Zip genes from various plant species are involved in diverse biological functions, including developmental processes in apical meristems and response to light, water stress or ABA (Ariel *et al.*, 2007; Henriksson *et al.*, 2005). The class-III HD-Zip proteins PHABULOSA and PHAVOLUTA have roles in determining radial patterning in shoots (McConnell *et al.*, 2001), and they are targeted by microRNAs (Mallory *et al.*, 2004). AtHB-1 is reported to function as a transcription activator, and affects leaf cell fate when overexpressed in tobacco (Aoyama *et al.*, 1995). More recently, *HaHB-4*, a class-I HD-Zip gene from sunflower transcriptionally regulated by water availability and ABA, has been reported as a new component of ethylene signalling pathways. Transgenic Arabidopsis plants overexpressing this gene have been shown to exhibit a marked delay in senescence, and are less sensitive to ethylene (Manavella *et al.*, 2006). Expression of this gene has a major repressive effect on genes related to ethylene synthesis, such as *ACO*, and on genes related to ethylene signalling, such as *ERF2* and *ERF5* (Manavella *et al.*, 2006). We report here on the identification of a cDNA clone for a previously uncharacterized HD-Zip protein from tomato, LeHB-1, that binds to *LeACO1* promoter fragments containing the putative HD protein binding sequences. Virus-induced silencing of *LeHB-1* was shown to inhibit *LeACO1* mRNA accumulation and ripening, whereas ectopic overexpression of the gene led to altered flower organ identity and conversion of sepals to fruit-like structures.

## Results

### LeHB-1 encodes an HD-Zip protein

**Figure 1:**
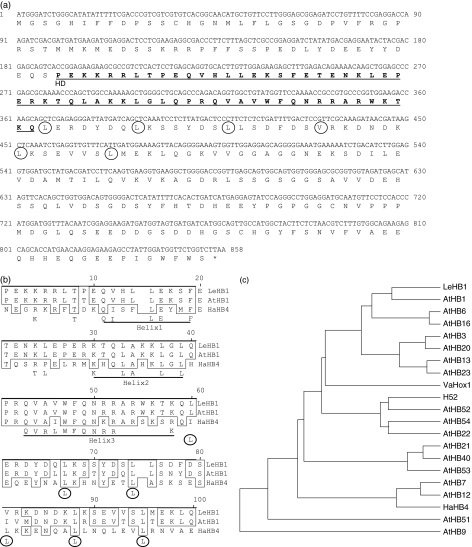
Sequence analysis of *LeHB-1*. (a) *LeHB-1* nucleotide and deduced amino acid sequences. The homeodomain (HD) is underlined and the conserved leucine residues are circled. (b) Comparison of LeHB-1 HD-Zip amino acid sequences with those from *Arabidopsis* AtHB-1 and Sunflower HaHB-4. Conserved regions are boxed. Conserved sequences in the HD are indicated below the alignment, and the three α helices of the HD and the leucine (or valine) residues are underlined. (c) A phylogenetic tree generated using the class-I HD-Zip protein sequences from Arabidopsis, HaHB-4 from sunflower, VaHox1 from tomato, and H52 and LeHB-1. AtHB-9 (Ariel *et al.*, 2007), a class-III HD-Zip protein from Arabidopsis, was used as an outgroup.

Important regulatory regions in the ACO1 promoter were identified previously by testing promoter-reporter gene constructs in transgenic plants (Blume and Grierson, 1997). Putative AtHB-1 binding sites were found subsequently in the *LeACO1* promoter, and a BLAST search of tomato expressed sequence tag (EST) databases was performed using the *AtHB-1* cDNA sequence. Tomato *LeHB-1* was identified as the closest match to *AtHB-1*, and we isolated the corresponding EST clone (tomato EST: TC183162), referred to here as *LeHB-1*, by RT-PCR using the primers LeHB-1F1/LeHB-1R1 corresponding to the coding sequence (Table 1). Sequencing confirmed that *LeHB-1* encodes a 285-aa protein (Figure 1a) with the conserved HD (aa 64–122) and Zip domains (aa 123–165). Sequence alignment indicated that LeHB-1 shares an overall 69% similarity to AtHB-1 (data not shown), and that its HD-Zip domains share 92 and 56% amino acid similarity to those of AtHB-1 and HaHB-4, respectively (Figure 1b). The conserved residues for the HD transcription factors are also present in the three proteins (Figure 1b). There are 17 class-I HD-Zip proteins in Arabidopsis (Henriksson *et al.*, 2005), and 15 have been found in the tomato databases so far (data not shown). Only a small number of them, however, have been characterized. Phylogenetic analysis using the full-length sequences of Arabidopsis class-I HD-Zip proteins, the sunflower HaHB-4, and two tomato class-I HD-Zip proteins VaHox1 and H52 (Mayda *et al.*, 1999; Tomero *et al.*, 1996), together with LeHB-1, indicated that among all these sequences LeHB-1 is most similar to AtHB-1 (Figure 1c).

**Table 1:** Primers used for this study

Primer	Sequences (5′→3′)	Gene or fragment amplified
LeHB1F1	ATGGGATCTGGGCATATA	LeHB-1 coding sequence
LeHB1R1	TTAAGACCAGAACCATCC	LeHB-1 coding sequence
LeHB15UTRf	CGCCCTCGCCGGAATCTTA	*LeHB-1* 5′-UTR
LeHB15UTRr	TCGCCTATTTACACCACGAAG	*LeHB-1* 5′-UTR
LeHB1F-Nhe	GCGCTAGCATGATGAAGATGGAGGAC	LeHB-1 HD-Zip
LeHB1R-Nhe	GCGCTAGCTTATCCCCCTGCTCCTCCAAC	LeHB-1 HD-Zip
F1-1F	CCTAACAGAGTTCGATGGGTT	*LeACO1* promoter F1-1
F1-1R	GGTGGAATAATTTGAAATAT	*LeACO1* promoter F1-1
F3-1F	ATTCAATAATGGAGTCAGGTG	LeACO1 promoter F3-1
F3-1R	ACCTCTCTGAAACAATTTCTCC	LeACO1 promoter F3-1
F4-1F	CATCTCAAATAATATTGAGTT	*LeACO1* promoter F4-1
F4-1R	AGAGTCCTAAACTTTTTCCTACC	*LeACO1* promoter F4-1
PP82(PVXF)	CAGTGTTGGCTTGCAAACTAG	Viral transient *LeHB-1*
PP228(PVXR)	GGGTAAGTTTTCCGTATGTTG	Viral transient *LeHB-1*
PP402	AGTTGA*Atcg****AT*G**GGATCTGGGCATATATTTTTC	Wild-type *LeHB-1*
PP403	AAATT*gggccc*AGACCAGAACCATCCAATAGGCTC	Wild-type/mutant *LeHB-1*
PP483	AGTTGA*Atcg****AT*c**tGATCTGGGCATATATTTTTC	Mutant *LeHB-1*
AC1897f	TCTCTTCAATCTTTTGTATTC	*LeACO1*
AC2540r	GTACTTGAGAGATATTAGAAGTAG	*LeACO1*

Introduced *Cla*I and *PspOM*I sites are set in *italic*, changed nucleotides are set in lower case letters, the start codon and its mutated version are set in **bold** font and the introduced stop codon is underlined.

### LeHB-1 is highly expressed in flowers and developing fruits

**Figure 2:**
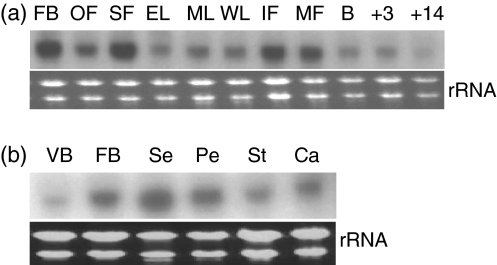
Northern analysis of *LeHB-1* mRNA. (a) Expression of *LeHB-1* in different tissues. Abbreviations: FB, flower buds; OF, fully open flowers; SF, senescing flowers; EL, emerging leaves; ML, mature leaves; WL, wounded mature leaves; IF, immature green fruit; MF, mature green fruit; Br, fruit at start of colour change; +3 and +14, 3 or 14 days after start of colour change. (b) Expression of *LeHB-1* in floral organs. Abbreviations: VB, vegetative buds; FB, floral buds; Se, sepals; Pe, petals; St, stamens; Ca, carpels. RNA (10 μg) was used for the northern blot. The full-length coding sequence of LeHB-1 was used as the probe. Ethidium bromide stained rRNA (rRNA) indicates equal loading.

Northern blots revealed that *LeHB-1* was highly expressed in tomato flower buds, senescing flowers (developing ovary stage), and developing immature and mature green fruits, but that the mRNA declined during ripening and was maintained at a stable but relatively low level in ripe fruits (Figure 2a). The *LeHB-1* transcripts were also expressed in emerging young leaves and fully-expanded mature leaves, but wounding had no effect on expression (Figure 2a). *LeHB-1* mRNA was much higher in flower buds in comparison with vegetative buds, and was abundant in all floral parts, particularly the sepals and the carpels (Figure 2b).

### LeHB-1 binds to the LeACO1 promoter *in vitro*

**Figure 3:**
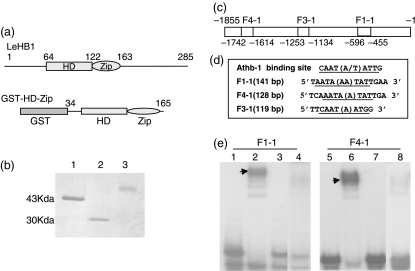
Binding of LeHB-1 to *LeACO1* promoter fragments. (a) Outlines of the LeHB-1 protein and GST-HD-Zip fusion protein used for DNA binding. (b) Expression of fusion protein in yeast. Lane 1, GST-LeHB1 HD-Zip fusion; lane 2, GST; lane 3, 1 μg BSA. (c) Structure of the *LeACO1* promoter showing the three regions used for LeHB-1 binding (F4-1, F3-1 and F1-1). (d)Comparison of AtHB-1 binding sequence with similar sequences found in F1-1, F4-1 and F3-1 of the *LeACO1* promoter. (e) Gel retardation assays with F1-1 and F4-1 fragments, and the LeHB-1 GST-HD-Zip fusion protein. Lane 1, free F1-1 probe; lane 2, GST-HD-Zip with F1-1 probe; lane 3, GST with F1-1 probe; lane 4, sample from lane 2 plus a 200-fold molar excess of unlabelled competitor F1-1; lane 5, free F4-1 probe; lane 6, GST-HD-Zip with the F4-1 probe; lane 7, GST with the F4-1 probe; lane 8, sample from lane 6 plus a 200-fold molar excess of unlabelled competitor F4-1.

To test whether the LeHB-1 protein had the capacity to bind the *LeACO1* promoter, we expressed the LeHB-1 HD-Zip polypeptide tagged with glutathione S-transferase (GST) (GST::HD-Zip) (Figure 3a) in *Saccharomyces pombe*, as the HD-Zip domains have been shown to be essential for the *in vitro* DNA binding activity of AtHB-1 (Sessa *et al.*, 1993). The free GST and GST::HD-Zip fusion proteins were then purified, and their integrity and purity were examined by SDS-PAGE (Figure 3b).

**Figure 4:**
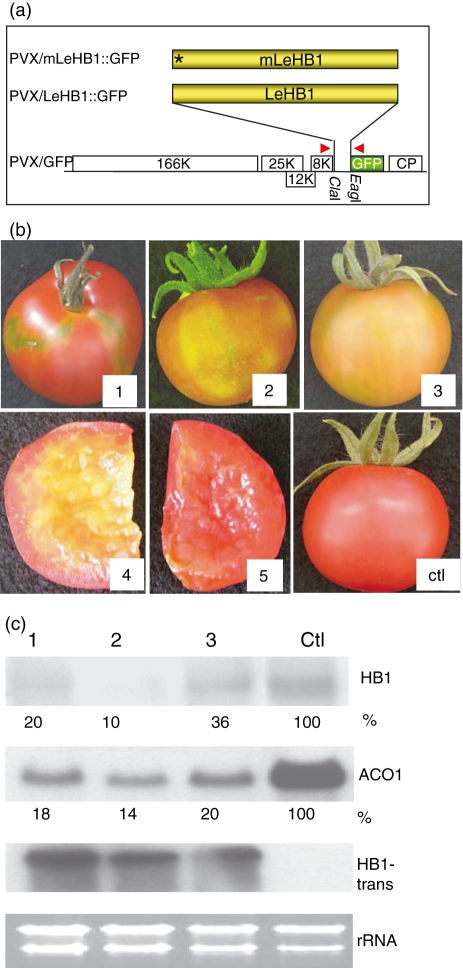
Effects of silencing *LeHB-1* on ripening and *LeACO1* expression. (a) The PVX/GFP vector (Wezel *et al.*, 2002) is shown together with the wild-type *LeHB-1* and the mutated *LeHB-1* (asterisked; Table 1) genes used to create PVX/LeHB1::GFP and PVX/mLeHB1::GFP, respectively. The PVX 166K-RDRP, movement proteins (25, 12 and 8K) and coat protein (CP) are indicated. The triangles show the positions of primers (Table 1) for detecting the transgene. (b) Fruit injected with PVX/LeHB1::GFP or PVX/mLeHB1::GFP (panels 1–4) and fruit injected with PVX/GFP (panels 5, Ctl). Photographs were taken 4 weeks post-injection. (c) Silencing endogenous *LeHB-1* downregulated *LeACO1* in virus-induced gene silencing (VIGS) fruits. Total RNA (10 μg) from delayed-ripening fruits injected with PVX/LeHB1::GFP or PVX/mLeHB1::GFP (from panels 1, 2 and 3) (lanes 1–3) and PVX/GFP control fruit (lane ctl) were used for northern analysis using the 5′-UTR of *LeHB-1* (HB1) and the first exon of *LeACO1* (ACO1) as probes. The quantification of *LeHB-1* and *LeACO1* mRNA by ^32^P radioactivity emission is given as a percentage. Viral transient *LeHB-1* (HB1-trans) in the PVX genome and subgenomes was detected in PVX/LeHB1::GFP- or PVX/mLeHB1::GFP-injected fruits (lanes 1–3), but not in control fruits (lane Ctl). Ethidium bromide stained rRNA (rRNA) indicates RNA loading.

Three *LeACO1* promoter regions (F1-1, F3-1 and F4-1), containing predicted homeobox *cis*-elements similar to the AtHB-1 binding sequence (CAATA/TATTG) (Figure 3c), were selected for the GST::HD-Zip/DNA binding analysis. The 141-bp F1-1 promoter fragment contains a 10-bp sequence AATA(AA)TATT with dyad symmetry, F3-1 (119 bp) has a 9-bp sequence CAAT(A)ATGG, and F4-1 (128 bp) has a 9-bp sequence AATA(A)TATT with dyad symmetry (Figure 3c). These three fragments were PCR amplified and sequenced. Incubation of GST::HD-Zip with either F1-1 or F4-1 resulted in a DNA–protein complex that showed an electrophoretic mobility shift compared with free DNA fragments (Figure 3d). The formation of the DNA–protein complex was specific, and was out-competed by a 200-fold molar excess of unlabelled starting DNA of each respective promoter region. However, the GST::HD-Zip fusion did not produce a similar complex with the fragment F3-1 (data not shown). No DNA–protein complex was formed between any of the promoter fragments and free GST (Figure 4d). These findings demonstrate that the LeHB-1 HD-Zip is capable of binding to the *LeACO1* promoter, probably by recognizing the 9 or 10-bp DNA sequences with dyad symmetry, indicating that LeHB-1 might be involved in transcriptional regulation of *LeACO1*.

### Virus-induced gene silencing (VIGS) of LeHB-1 delays ripening

To determine whether LeHB-1 regulated *LeACO1* gene expression *in vivo*, we attempted to overexpress or silence *LeHB-1* in stably transformed tomato plants using the constitutive 35S promoter, but this failed, indicating that *LeHB-1* overexpression or knock-down might be deleterious or lethal. We then employed VIGS (Manning *et al.*, 2006) to inhibit the *LeHB-1* gene. The cDNA encoding the full-length LeHB-1 protein, and a non-sense mutant derivative with Met^1^ to Ile followed by a stop codon, were PCR-amplified and cloned into the potato virus X/Green fluorescent protein (PVX/GFP) vector to generate PVX/LeHB1::GFP and PVX/mLeHB1::GFP constructs (Table 1, Figure 4a and Experimental procedures). Viral RNA transcripts were needle-injected into the carpopodium of wild-type Ailsa Craig tomato fruits attached to the plant, and the effects on the fruit were observed 2–4 weeks later. Thirty fruits (out of approximately 90) injected with PVX/LeHB1::GFP or PVX/mLeHB1::GFP produced regions that failed to ripen normally and displayed a distinct green sector, indicative of delayed ripening (Figure 4b, panel 1), or produced yellow (partially ripened) fruits (Figure 4b, panels 2, 3). The green sectors of inhibited ripening on fruit of VIGS LeHB-1 plants eventually turned orange, and showed signs of slow ripening. All control fruits injected with the PVX/GFP vector ripened normally (Figure 4b, Ctl panel). Strikingly, some of the delayed-ripening phenotypes (Figure 4b, panels 2, 3) mimicked the fruits produced by *LeACO1* antisense transgenic plants, in which *LeACO1* mRNA was inhibited by 95% (Picton *et al.*, 1993).

Northern analysis of total RNA isolated from the VIGS delayed-ripening fruits and the control fruits, at 7 days after the start of colour change, showed that the endogenous *LeHB-1* expression was 64–90% downregulated in the delayed-ripening fruits and fruit sectors, compared with the controls (Figure 4c), and that *LeACO1* expression was also reduced by 80–86% in the fruits where ripening was delayed by VIGS (Figure 4c), indicating a mechanistic link exists between LeHB-1, *LeACO1* and ripening. The viral delivery of the silencing inducer, i.e. the wild-type or the mutated *LeHB-1* RNA, was readily detected in the VIGS fruits, but not in the control PVX/GFP (Figure 4c, HB1-tran). Taken together, this evidence supports the suggestion that LeHB-1 functions as a transcription activator in the regulation of *LeACO1* expression and ripening.

### Ectopic expression of LeHB-1 disrupts flower development

**Figure 5:**
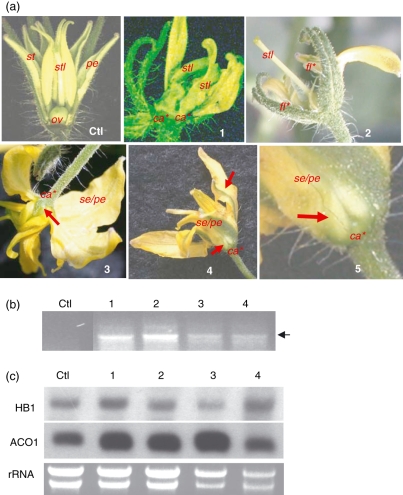
Effects of viral transient expression of LeHB-1 on flower development. (a) Phenotypes of the flowers. Ctl: control flower with part of the calyx and corolla removed. Panel 1: double carpel-like structures (ca*) with swollen styles (stl) that developed in place of the ovary. Panel 2: triple flowers (fl*) within one original flower – the pistil of the central flower is flanked by two additional flowers, and all are enclosed by one whorl of sepals. Panels 3 and 4: fusion of sepal (green) and petal (yellow) (*se/pe*) with swollen green structure at the base of the sepal (ca*). Panel 5: 5× enlarged image of panel 4, showing the swollen carpel-like structure on the sepal. (b) RT-PCR detection of viral delivery of *LeHB-1* (arrow) using the primers corresponding to the pVX vector (pp82/pp228, Table 1) with RNA extracted from the abnormal flowers 1–4 infected with PVX/LeHB-1::GFP in (a), and a non-infected control flower (Ctl). (c) Northern analysis of endogenous *LeHB-1* and *LeACO1* expression using RNA samples isolated from abnormal flowers 1–4 infected with PVX/LeHB-1::GFP in (a), and a non-infected control flower (Ctl), using the LeHB-1 5′ untranslated region (HB1) and the first exon of LeACO1 (ACO1) as probes. Ethidium bromide stained rRNA (rRNA) shows sample loading (lanes Ctl, 1, 2, 10 μg total RNA; lanes 3–4, 5 μg total RNA).

Introduction of recombinant viruses into plants can, in addition to producing local effects, also lead to systemic movement and expression of virus genes elsewhere in developing plants (Chapman *et al.*, 1992). Viral delivery of wild-type LeHB-1 to distant flowers sometimes altered floral organ identity, and caused remarkable flower developmental abnormalities. This was associated with overexpression, not silencing, of the virus-delivered *LeHB-1* gene. Flower abnormalities included production of multiple flowers or carpel-like structures within one sepal whorl (Figure 5a, panels 1, 2), fused sepals and petals (Figure 5a, panels 3, 4), and swelling of the base of the sepals (Figure 5a, panels 3, 4, 5). Delivery of the *LeHB-1* transgene to the abnormal flowers and its transcription were confirmed by RT-PCR using the primers corresponding to the PVX vector (Figure 5b and Table 1). Furthermore, the endogenous *LeHB-1* mRNA in these samples was readily detectable and showed no obvious reduction compared with the control [Figure 5(c); note that 10 μg of total RNA was used for the control, and for lanes 1 and 2, and that 5 μg was used in lanes 3 and 4]. These results indicated that overexpression of LeHB-1 was occurring in these organs. In addition, the *LeACO1* transcripts were more abundant in the abnormal flowers than in the control (Figure 5c), indicating that ectopic expression of *LeHB-1 in vivo* enhanced the accumulation of *LeACO1* transcripts above their normal level. The fact that these flower phenotypes were not found in the plants infected with the PVX/GFP vector, or the mutated PVX/mLeHB::GFP construct (data not shown), suggested that functional LeHB-1 was required to cause these effects.

### Ectopic expression of LeHB-1 converts sepals into fruits

**Figure 6:**
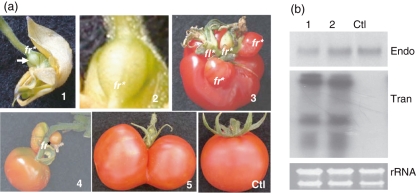
Development of carpel-like structures from sepals in fruit of PVX/LeHB1::GFP-injected plants. (a) Panel 1: a fruit-like structure (fr*, arrow) arising from the sepal of a PVX/LeHB-1::GFP-infected flower. Panel 2: 5× enlarged image of panel 1. Panel 3: multiple ripe fruit-like structures (fr*) and flowers (fl*) developed from the sepals of one original, much larger, fruit after injection with PVX/LeHB-1::GFP. Panel 4: a second fruit developed from the elongated pedicel of the original fruit following injection with PVX/LeHB-1::GFP. Panel 5: twin fruits developed from one pedicel injected with PVX/LeHB-1::GFP. Ctl: control fruit injected with PVX/GFP. (b) Northern analysis of endogenous *LeHB-1* (Endo) and the *LeHB-1* transgene mRNA (Trans) in abnormal and control fruits. RNA samples were isolated from the lower part of the fruit, shown in panel 1 (lane 1), or the upper parts of the same fruit, including the mini fruits and the floral structures, shown in panel 3 (lane 2), and control fruit. Ethidium bromide stained rRNA (rRNA) shows RNA loading.

Viral delivery of the wild-type *LeHB-1* construct into flowers also triggered the production of swollen green structures in the position of the sepals. In several instances, conversion of sepals into carpel-like structures was evident (Figure 6a, panels 1, 2), and these eventually developed into fruit-like structures and ripened (Figure 6a, panels 3, 4). Sometimes twin fruits were produced, or an additional fruit developed from one pedicel (Figure 6a, panels 4, 5). None of these phenotypes was seen in the plants infected with the mutated *LeHB-1* construct, the PVX vector control or in non-infected plants. Northern analysis indicated that the *LeHB-1* transgene mRNA was detected in the abnormal fruits but not in the control fruit, and that the endogenous *LeHB-1* was still expressed (Figure 6b). The results again suggested that these phenotypes were caused by overexpression of the full-length *LeHB-1* gene *in planta*.

## Discussion

This study demonstrates that LeHB-1 is involved in the regulation of tomato floral organogenesis, carpel development and ripening. LeHB-1 encodes a class-I HD-Zip protein that binds to the promoter of *LeACO1* (Figures 1, 3). VIGS silencing of LeHB-1 results in a significant delayed-ripening phenotype, which is associated with a great reduction of *LeACO1* mRNA (Figure 4). Antisense inhibition of *LeACO1* has been shown previously to lead to reduced ethylene synthesis (Hamilton *et al.*, 1990; Picton *et al.*, 1993). These results are consistent with the suggestion that LeHB-1 positively controls *LeACO1*, and that silencing LeHB-1 represses *LeACO1*, which consequently leads to delayed ripening. Putative LeHB-1 binding sites are also found in the promoters of a number of ripening related genes, including, LeACO2, PG1, LeMADS-RIN and NAC-NOR (ZL and DG, unpublished data), and it is possible that LeHB-1 may directly regulate these ripening-related genes. The identification of LeHB-1 marks a further step in our understanding of ripening control, and begins to answer a long-standing and key question about how *LeACO1* is regulated. *LeHB-1* is highly expressed in mature green fruit and declines at the breaker stage, whereas *LeACO1* mRNA increases in mature green fruit and accumulates during ripening (Blume and Grierson, 1997). This difference might be explained by differences between mRNA accumulation and protein turnover, but could also suggest that other factors, in addition to LeHB-1, control LeACO1 mRNA accumulation.

Homeobox genes were originally discovered in *Drosophila*, and were shown to function as transcriptional regulators that control embryonic morphogenesis. They regulate diverse aspects of morphogenesis (Graba *et al.*, 1997), and are now known to play a role in the control of hormones in plants and animals (Sakamoto *et al.*, 2001; Susa *et al.*, 2007). The first plant homeobox gene to be identified was *KNOTTED 1* (Vollbrecht *et al.*, 1991), and this and related genes are involved in the control of GA and cytokinin (Hay *et al.*, 2002; Jasinski *et al.*, 2002; Ori *et al.*, 1999; Sakamoto *et al.*, 2001). Several HD-Zip genes have also been implicated in the control of, or responses to, other hormones, such as ABA (Ditzer and Bartels, 2006; Lee *et al.*, 2001; Söderman *et al.*, 1996, 1999), auxin (Baima *et al.*, 1995; Plesch *et al.*, 1997), red/far-red light effects on cell expansion (Carabelli *et al.*, 1996; Steindler *et al.*, 1999), de-etiolation (Aoyama *et al.*, 1995) and blue-light signalling (Wang *et al.*, 2003). The sunflower HD-Zip gene *HaHB-4*, which is induced by ABA, has been implicated in senescence and ethylene signalling (Manavella *et al.*, 2006), but is distinct from LeHB-1 (Figure 1). The present studies demonstrate that LeHB-1 is not only involved in ethylene and ripening, but also in flower and fruit development.

Altered floral organ identity (Figure 5) and conversion of the sepals into carpel-like structures (Figure 6), caused by transient overexpression of LeHB-1 *in planta*, highlight a crucial role for LeHB-1 in floral organogenesis, which is consistent with the abundance of *LeHB*-1 transcripts in floral organs and developing fruits (Figure 2). Ripening-related changes in sepals can be induced by low temperature in VFNT cherry tomatoes (Bartley and Ishida, 2003). The cells swell, turn red, express ripening-related genes (Bartley and Ishida, 2003) and accumulate mRNA for transcription factors TAG1, TMG, LeAP2 and VaHox1 (Bartley and Ishida, 2007). Interestingly, ethylene is required in order for mRNA accumulation, except for VaHox1, a tomato class-I HD-Zip gene (Tomero *et al.*, 1996; Figure 1). A role for ethylene in flower and fruit development has been suggested by previous studies. For example, ethylene is known to stimulate female flower development in cucumber (Yamasaki *et al.*, 2003), and to be central to the conversion of vegetative to floral buds in Arabidopsis (Achard *et al.*, 2007). Ethylene biosynthesis also increases in carnation styles and ovaries after pollination (Jones and Woodson, 1997), and in tomato, there is evidence for ethylene involvement in carpel development (Llop-Tous *et al.*, 2000). In the normal course of events, it is possible that, in addition to its regulation of *LeACO1*, LeHB-1 is involved in the control of flower development through a network of regulatory factors, including MADS-box genes (Becker and Theißen, 2003) and perhaps other HD-Zip genes. In the present experiments, ectopic overexpression of LeHB-1 altered floral organ identity and triggered the formation of carpel-like structures from sepals, which was associated with increased accumulation of *LeACO1* mRNA (Figure 5). It seems unlikely that enhanced *LeACO1* mRNA alone could result in these developmental changes, as they are not found when *LeACO1* is overexpressed in tomato under the control of the 35S promoter (DG and Y. Han, unpublished data), or in plants overexpressing ACC synthase (H. Klee, personal communication). It is proposed that the unscheduled synthesis of LeHB-1 in cells of floral organs affects a series of genes that leads to altered floral development and ectopic carpel formation. It has yet to be established whether this occurs during normal development or only as a result of ectopic overexression of LeHB-1.

This study provides evidence for the control of the hormone ethylene by an HD-Zip homeobox gene, and suggests a link between LeHB-1 and the regulation of floral organogenesis, fruit development and ripening. Identification of the targets for LeHB-1 should generate new insights into the hormonal control of floral organ identity and early fruit development.

## Experimental procedures

### Preparation of the GST::LeHB-1 HD-Zip fusion protein

The partial LeHB-1 cDNA encoding the combined homeobox and leucine zip domain (HD-Zip, aa 34–165) was amplified by PCR using a pair of primers LeHB1F-Nhe and LeHB1R-Nhe (Table 1). The PCR product was digested with *Nhe*I and cloned into the *Nhe*I site of vector pESP-2 (Stratagene, http://www.stratagene.com). The construct was confirmed by sequencing and then transformed into yeast *S. pombe* strain SP-Q01. The fusion protein GST::HD-Zip together with the free GST protein were purified on GST affinity resin following the manufacturer's instructions (Stratagene), and were then examined by SDS-PAGE and visualized by Coomassie blue staining (CBB R250).

### *In vitro* gel retardation assay

The *LeACO1* promoter fragments F1-1, F3-1 and F4-1 were amplified by PCR using various sets of specific primers (Table 1), cloned into pGEM-T-Easy vector (Promega, http://www.promega.com) and sequenced. Radiolabelled fragments were prepared using the Rediprime II random prime labelling system (Amersham Pharmacia Biotech, http://www.gelifesciences.com). Double-stranded ^32^P-labelled DNA (3 ng) was incubated with 1 μg purified GST::HD-Zip fusion protein or GST in binding buffer (10 mm Tris, pH 7.5, 50 mm NaCl, 1 mm DTT, 2 mm EDTA), which contained 1 μg of double-stranded poly (dI:dC) (Amersham Pharmacia Biotech). Binding reactions were incubated in a volume of 20 μl for 0.5 h at 4°C, with gentle shaking. After incubation, the mixture was immediately loaded on to 4.5% polyacrylamide gels. Electrophoresis was carried out in 0.5 × TAE (2.42 g Tris base, 0.571 ml glacial acetic acid and 1 ml 0.5 m EDTA, pH 8.0, per litre) for 2.5 h at 14 mA, the gel was then dried and subjected to autoradiography at −80°C.

### RNA isolation and northern analysis

Fruit RNA extraction and northern blotting were carried out as described previously (Griffiths *et al.*, 1999). Hybridizations were carried out for 16 h at 42°C in buffer containing 1% (w/v) SDS, 50% (v/v) deionized formamide, 5 × SSC, 50 mm sodium phosphate, pH 6.8, 0.1% (w/v) sodium pyrophosphate, 10% (w/v) dextran sulphate and 50 mg ml^−1^ salmon sperm DNA. Radiolabelled probes were prepared using the Rediprime II random prime labelling system (Amersham Pharmacia Biotech). Hybridized membranes were finally washed in 0.2 × SSC, 0.1% SDS, and autoradiography was used to detect the signal. Quantitative analysis of the northern blots was carried out by ^32^P radioactivity emission using the Personal Molecular Imager FX system (Bio-Rad, http://www.bio-rad.com) following the manufacturer's instructions.

### RT-PCR

Flower RNA was isolated using the RNeasy plant mini kit (Qiagen, http://www.qiagen.com) following the manufacturer's instructions. A 2-μg portion of total RNA was used for reverse transcription in a reaction volume of 20 μl using SuperScript™ II Reverse Transcriptase (Invitrogen, http://www.invitrogen.com). A 2-μl volume of this RT mixture was then used for PCR using primers pp82/pp228 (Table 1).

### VIGS and viral-transient gene expression

The wild-type and mutant *LeHB-1* gene sequences were PCR amplified using the *pfu* DNA polymerase (Promega), and a set of primers PP402/PP403 (wild type) or PP483/PP403 (mutant) (Table 1), respectively. The resultant PCR products were digested with *PspOM*I and *Eag*I, and were then cloned into the *Cla*I/*Eag*I sites in frame with the GFP sequences in the PVX/GFP vector (Wezel *et al.*, 2002), to produce PVX/LeHB1::GFP and PVX/mLeHB1::GFP. RNA transcripts produced by *in vitro* transcription of PVX/LeHB1::GFP, PVX/mLeHB1::GFP and PVX/GFP, after linearization with *Spe*I, were needle-injected into the carpopodium of young tomato fruits (*Lycopersicon esculentum* cv. Ailsa Craig) attached to the plant (Manning *et al.*, 2006). Injected fruits ranged from 2 mm to 4 cm in size on different trusses on the same plant, and on different plants. Within 2–4 weeks after injection, the VIGS-mediated effects on tomato ripening appeared. These and subsequent effects of viral transient *LeHB-1* gene expression on flower and fruit development were then routinely examined, and were photographically recorded with a Nikon Coolpix 995 digital camera.
